# Effect of Hydrogen Peroxide and Superoxide Anions on Cytosolic Ca^2+^: Comparison of Endothelial Cells from Large-Sized and Small-Sized Arteries

**DOI:** 10.1371/journal.pone.0025432

**Published:** 2011-09-26

**Authors:** Lei Sun, Ho-Yan Yau, On-Chai Lau, Yu Huang, Xiaoqiang Yao

**Affiliations:** School of Biomedical Sciences and Li Ka Shing Institute of Health Sciences, The Chinese University of Hong Kong, Hong Kong, China; University of Arizona, United States of America

## Abstract

We compared the Ca^2+^ responses to reactive oxygen species (ROS) between mouse endothelial cells derived from large-sized arteries, aortas (aortic ECs), and small-sized arteries, mesenteric arteries (MAECs). Application of hydrogen peroxide (H_2_O_2_) caused an increase in cytosolic Ca^2+^ levels ([Ca^2+^]_i_) in both cell types. The [Ca^2+^]_i_ rises diminished in the presence of U73122, a phospholipase C inhibitor, or *Xestospongin* C (XeC), an inhibitor for inositol-1,4,5-trisphosphate (IP_3_) receptors. Removal of Ca^2+^ from the bath also decreased the [Ca^2+^]_i_ rises in response to H_2_O_2_. In addition, treatment of endothelial cells with H_2_O_2_ reduced the [Ca^2+^]_i_ responses to subsequent challenge of ATP. The decreased [Ca^2+^]_i_ responses to ATP were resulted from a pre-depletion of intracellular Ca^2+^ stores by H_2_O_2_. Interestingly, we also found that Ca^2+^ store depletion was more sensitive to H_2_O_2_ treatment in endothelial cells of mesenteric arteries than those of aortas. Hypoxanthine-xanthine oxidase (HX-XO) was also found to induce [Ca^2+^]_i_ rises in both types of endothelial cells, the effect of which was mediated by superoxide anions and H_2_O_2_ but not by hydroxyl radical. H_2_O_2_ contribution in HX-XO-induced [Ca^2+^]_i_ rises were more significant in endothelial cells from mesenteric arteries than those from aortas. In summary, H_2_O_2_ could induce store Ca^2+^ release via phospholipase C-IP_3_ pathway in endothelial cells. Resultant emptying of intracellular Ca^2+^ stores contributed to the reduced [Ca^2+^]_i_ responses to subsequent ATP challenge. The [Ca^2+^]_i_ responses were more sensitive to H_2_O_2_ in endothelial cells of small-sized arteries than those of large-sized arteries.

## Introduction

Vascular endothelial cells *in vivo* are constantly exposed to ROS that are released from neutrophils, macrophages, and vascular smooth muscle cells [1], [2]. Moreover, endothelial cells themselves are generators of ROS [1], [2]. The main ROS that are produced include superoxide anions, H_2_O_2_, hydroxyl radicals and peroxynitrite. Functionally, ROS play a key role in physiological and pathological processes in endothelial cells. For example, H_2_O_2_ at physiological concentration serves as an endothelium-derived hyperpolarizing factor (EDHF), mediating vascular relaxation [3]. However, excessive production of ROS causes extensive damage to the structure and function of endothelial cells, leading to endothelial dysfunction [1]. Evidence indicates that ROS-induced endothelial function and dysfunction are often preceded by an alteration in endothelial [Ca^2+^]_i_ [4], which serves as an important second messenger to induce diverse responses.

Reports showed that superoxide anions [5], H_2_O_2_ [6]–[9], and hydroxyl radical [5], [10] are all capable of inducing [Ca^2+^]_i_ rises in vascular endothelial cells. The [Ca^2+^]_i_ rises could result from ROS actions on the plasma membrane ion channels [11], IP_3_ production [7], [12], IP_3_ receptors [13], [14], and/or endoplasmic reticulum Ca^2+^-ATPase [6]. In addition to their direct action on endothelial [Ca^2+^]_i_, ROS treatment may alter the [Ca^2+^]_i_ responses of endothelial cells to a variety of physiological agonists including ATP and bradykinin [7], [9], [12]. However, the results of these studies are often controversial. In some studies, ROS treatment was found to enhance the agonist-induced [Ca^2+^]_i_ rises [12], whereas in other studies ROS were found to attenuate [9], [15] or have no effect [7] on the agonist-induced [Ca^2+^]_i_ responses.

Although there have been a great number of studies investigating the ROS effect on [Ca^2+^]_i_ in endothelial cells, most of these reports only investigated the endothelial cells derived from large-sized arteries [5]–[10], [12], [15]. The role of ROS on [Ca^2+^]_i_ in endothelial cells of small-sized arteries has received little attention [but see 8]. It is unclear whether there is any difference in ROS-induced [Ca^2+^]_i_ responses in endothelial cells from different-sized arteries. Large-sized arteries and small-sized arteries differ in their function. Small-sized arteries such as mesenteric arteries are resistance arteries that play a key role in blood pressure control. Vasoactive factors in small-sized arteries are often different from that in large-sized arteries. For example, while nitric oxide is the major vasodilator in large arteries, EDHFs often play a more important role as vasodilators in small-sized arteries [16].

In the present study, we compared the effect of H_2_O_2_ on [Ca^2+^]_i_ in endothelial cells from large-sized arteries, aortas (aortic ECs), and small-sized arteries, mesenteric arteries (MAECs). We found that H_2_O_2_ stimulated IP_3_ production to induce store Ca^2+^ release in both cell types. H_2_O_2_ treatment depleted intracellular [Ca^2+^]_i_ stores, resulted in a decreased [Ca^2+^]_i_ response to subsequent ATP challenge. The Ca^2+^ store depletion was more sensitive to H_2_O_2_ in endothelial cells of small-sized arteries than those of large-sized arteries.

## Results

### Both Ca^2+^ entry and store Ca^2+^ release contributed to H_2_O_2_-induced [Ca^2+^]_i_ rises

**Figure 1:**
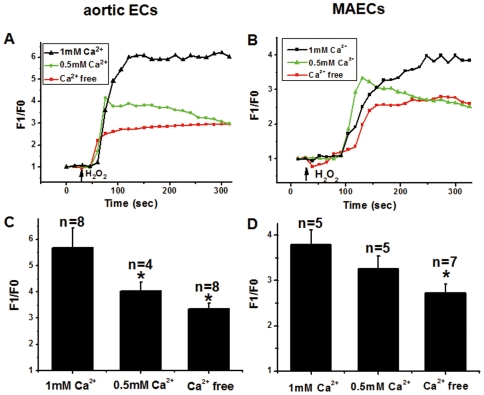
Effect of extracellular Ca^2+^ on H_2_O_2_-induced [Ca^2+^]_i_ rises in aortic ECs and MAECs. **A and B**. Representative traces of [Ca^2+^]_i_ rises in response to 5 mM H_2_O_2_ in the primary cultured aortic ECs (A) and MAECs (B) that were bathed in N-PSS (1 mM Ca^2+^), 0.5Ca^2+^-PSS (0.5 mM Ca^2+^) or 0Ca^2+^-PSS (nominal Ca^2+^-free). Fluorescence intensity before H_2_O_2_ application was normalized to 1 as F0. **C and D**. Summary of the maximal [Ca^2+^]_i_ changes to H_2_O_2_ as expressed in F1/F0. Mean±SEM of 4 to 8 independent experiments (10 to 15 cells per experiment). *, *P*<0.05 as compared to N-PSS.

The effect of H_2_O_2_ on [Ca^2+^]_i_ was investigated in aortic ECs and MAECs. H_2_O_2_ at 5 mM caused marked [Ca^2+^]_i_ rises in both types of cells that were bathed in normal physiological saline solution (N-PSS) containing 1 mM Ca^2+^ (Figure 1A–1D). The amplitude of [Ca^2+^]_i_ rises to H_2_O_2_ reduced when bath Ca^2+^ was decreased to 0.5 mM or to nominal Ca^2+^-free (0Ca^2+^-PSS), suggesting a contribution of Ca^2+^ entry to the H_2_O_2_-induced [Ca^2+^]_i_ rises. Significant [Ca^2+^]_i_ rises to H_2_O_2_ could still be observed even when bath was Ca^2+^-free, suggesting that store Ca^2+^ release also contributed to the H_2_O_2_-induced [Ca^2+^]_i_ rises.

### H_2_O_2_ enhanced IP_3_ production and store Ca^2+^ release

**Figure 2:**
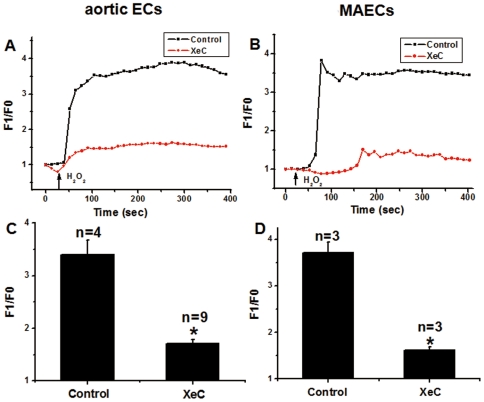
Effect of XeC on H_2_O_2_-induced [Ca^2+^]_i_ rises in aortic ECs and MAECs. **A and B**. Representative traces showing the [Ca^2+^]_i_ rises in response to 5 mM H_2_O_2_ with or without XeC. The cells were pre-treated with or without 10 µM XeC for 20 min in N-PSS before H_2_O_2_ challenge. Fluorescence intensity before H_2_O_2_ application was normalized to 1 as F0. **C and D**. Summary of data showing the effect of XeC (C and D) on H_2_O_2_-induced maximal [Ca^2+^]_i_ rises in aortic ECs (C) and MAECs (D) as expressed in F1/F0. Mean ± SEM of 3 to 9 independent experiments (10 to 15 cells per experiment). *, *P*<0.05 as compared to control.

**Figure 3:**
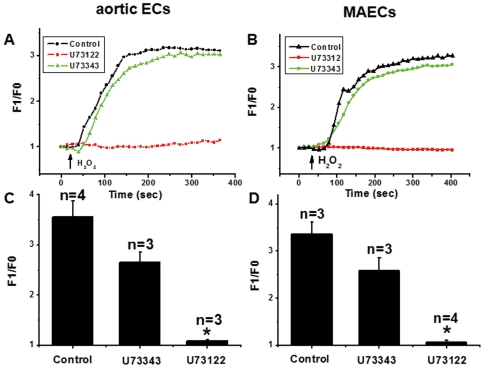
Effect of U73122 on H_2_O_2_-induced [Ca^2+^]_i_ rises in aortic ECs and MAECs. **A and B**. Representative traces showing the [Ca^2+^]_i_ rises in response to 5 mM H_2_O_2_ with or without U73122 or U73343. The cells were pre-treated with or without 10 µM U73122 or 10 µM U73343 for 30 min in N-PSS. Control had no U73122 and U73343. Fluorescence intensity before H_2_O_2_ application was normalized to 1 as F0. **C and D**. Summary of data showing the effect of 10 µM U73122 or 10 µM U73343 on H_2_O_2_-induced maximal [Ca^2+^]_i_ rises in aortic ECs (C) and MAECs (D) as expressed in F1/F0. Mean ± SEM of 3 to 4 independent experiments (10 to 15 cells per experiment). *, *P*<0.05 as compared to U73343.

**Figure 4:**
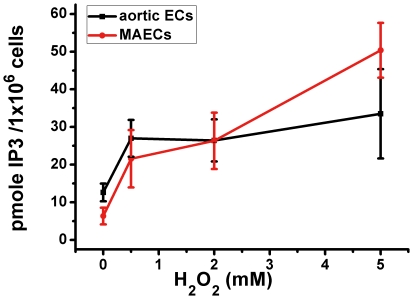
H_2_O_2_-induced IP_3_ production in a H_2_O_2_ concentration-dependent manner in aortic ECs and MAECs. The intracellular IP_3_ production was measured in aortic ECs and MAECs after different concentration of H_2_O_2_ challenge (500 µM, 2 mM and 5 mM), according to the protocols described in Methods. Mean±SEM of 3 independent experiments.

It is well documented that IP_3_-sensitive Ca^2+^ stores are the major intracellular Ca^2+^ stores, and that the Ca^2+^ release from the stores hinges on the production on IP_3_, which is generated through activity of phospholipase C (PLC) [17]. Figure 2A–2D show that treatment of the cells with XeC, an IP_3_ receptor inhibitor, at 10 µM for 20 min almost abolished the H_2_O_2_-induced [Ca^2+^]_i_ rises in both aortic ECs and MAECs. Furthermore, a PLC inhibitor U73122 (10 µM) markedly reduced the H_2_O_2_-induced [Ca^2+^]_i_ rises, whereas its inactive analog U73343 (10 µM) had no effect (Figure 3A–3D). These results suggest that the action of H_2_O_2_ mediated through IP_3_, which binds to IP_3_ receptors to release Ca^2+^ from intracellular Ca^2+^ stores. This was confirmed by experiments that measures IP_3_ production (Figure 4). Treatment of cells with H_2_O_2_ caused a H_2_O_2_ concentration-dependent increase in IP_3_ levels in both types of endothelial cells (Figure 4).

### H_2_O_2_ reduced the [Ca^2+^]_i_ responses to ATP in a H_2_O_2_ concentration and incubation time dependent manner

**Figure 5:**
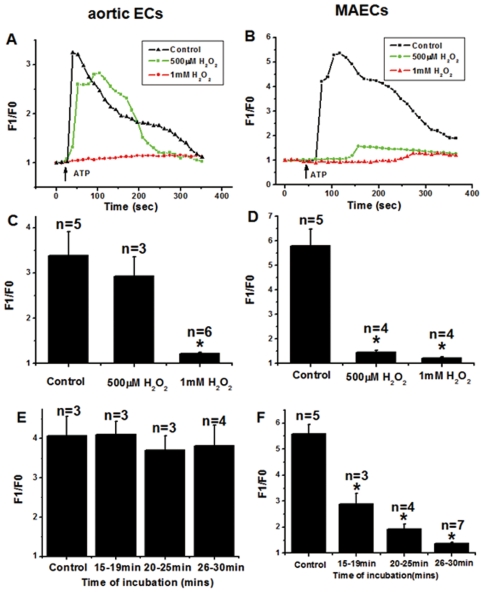
Effect of H_2_O_2_ pre-treatment on ATP-induced [Ca^2+^]_i_ rises in aortic ECs and MAECs. **A and B**. Representative traces showing the [Ca^2+^]_i_ rises in response to 30 µM ATP. The cells were pre-treated with or without H_2_O_2_ (500 µM or 1 mM as indicated) in N-PSS for 30 min, followed by ATP challenge. Control had no H_2_O_2_ treatment. Fluorescence intensity before ATP application was normalized to 1 as F0. **C and D**. Summary of data showing the ATP-induced maximal [Ca^2+^]_i_ rises as in A and B, expressed in F1/F0. **E and F.** Summary of data showing the ATP-induced maximal [Ca^2+^]_i_ rises after the cells were treated with 500 µM H_2_O_2_ for different period of time in N-PSS. Mean±SEM of 3 to 7 independent experiments (10 to 15 cells per experiment). *, *P*<0.05 as compared to control.

We next examined the effect of H_2_O_2_ treatment on agonist (ATP)-induced [Ca^2+^]_i_ rises. The cells were first pre-incubated with H_2_O_2_ (500 µM or 1 mM) for 30 min, followed by 30 µM ATP application to evoke [Ca^2+^]_i_ responses. Figure 5A and 5B show the representative traces of [Ca^2+^]_i_ rises in response to ATP in aortic ECs and MAECs that were pre-incubated with different concentrations of H_2_O_2_. A marked difference was found between aortic ECs and MAECs. While both cells lost the [Ca^2+^]_i_ responses to ATP after 1 mM H_2_O_2_ treatment, a relatively low concentration of 500 µM H_2_O_2_ could abolish the ATP responses in MAECs but had no effect in aortic ECs (Figure 5A–5D). To further confirm the difference between aortic ECs and MAECs, time series experiments were carried out. 500 µM H_2_O_2_ caused a time dependent decrease in the [Ca^2+^]_i_ responses to ATP in MAECs (Figure 5F) but not in aortic ECs (Figure 5E).

### H_2_O_2_ induced Ca^2+^ store depletion

**Figure 6:**
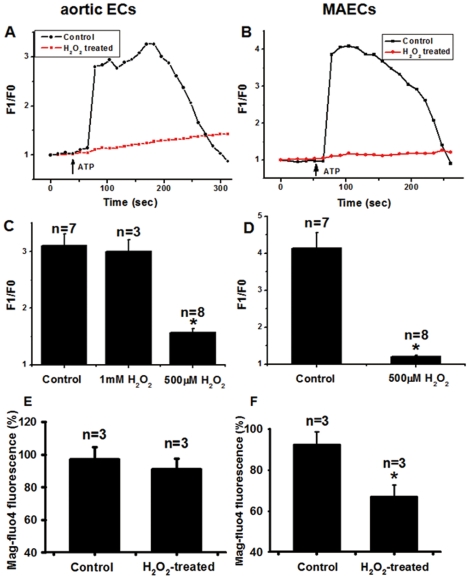
Depleting effect of H_2_O_2_ on store Ca^2+^ content in aortic ECs and MAECs. **A and B**. Representative traces showing the [Ca^2+^]_i_ rises in response to 30 µM ATP. The cells were pre-treated with or without 1 mM H_2_O_2_ for 30 min in N-PSS. Control had no H_2_O_2_ treatment. Cells were transferred to 0Ca^2+^-PSS and then challenged by ATP. Fluorescence intensity before ATP application was normalized to 1 as F0. **C and D**. Summary of data showing the effect of H_2_O_2_ (500 µM or 1 mM as indicated) on ATP-induced maximal [Ca^2+^]_i_ rises in aortic ECs and MAECs as expressed in F1/F0. **E and F**. Summary of data showing the effect of H_2_O_2_ treatment on store Ca^2+^ content as determined by Mag-fluo4 fluorescence in aortic ECs and MAECs. The cells were treated with or without 500 µM H_2_O_2_ for 30 min. Mean±SEM of 3 to 17 independent experiments (10 to 15 cells per experiment). *, *P*<0.05 as compared to control.

The reduced [Ca^2+^]_i_ responses to ATP could result from a decreased Ca^2+^ entry or a reduced Ca^2+^ release from intracellular Ca^2+^ stores. To focus on the store Ca^2+^ release alone, we next studied the ATP (30 µM)-induced [Ca^2+^]_i_ rises in cells bathed in a nominal Ca^2+^-free solution (Figure 6). Under this condition, [Ca^2+^]_i_ rises could only be attributed to the store Ca^2+^ release. The results show that ATP still triggered large [Ca^2+^]_i_ responses, which could be abolished by pre-treating aortic ECs for 25–30 min with 1 mM H_2_O_2_ but not 500 µM H_2_O_2_ (Figure 6A and 6C). For MAECs, treatment with a lower concentration (500 µM, 26–30 min) was enough to abolish the [Ca^2+^]_i_ responses to ATP (Figure 6B and 6D).

To further confirm the findings, Mag-fluo4/AM, a dye that stains Ca^2+^ in intracellular Ca^2+^ stores, was used to directly measure the store Ca^2+^ content. As shown in Figure 6E–6F, treatment with 500 µM H_2_O_2_ for 26–30 min caused a marked reduction of store Ca^2+^ content by 33±6% (n = 3) in MAECs but had no significant effect in aortic ECs. These data suggest that MAECs were more sensitive to H_2_O_2_ treatment than aortic ECs with regard to their responses in Ca^2+^ store depletion and ATP-induced [Ca^2+^]_i_ rises. The controls in Figure 6E and 6F were time controls, in which the cells went through 30 min incubation in the absence of H_2_O_2_. In time control, Mag-fluo4 fluorescence only decreased by 8±6% (n = 3) in MAECs and by 3±7% (n = 3) in aortic ECs. The small reduction in Mag-fluo4 fluorescence in the control experiments could be due to light-sensitive quenching of Mag-fluo4 as described elsewhere [18].

### [Ca^2+^]_i_ responses to ATP in the absence of H_2_O_2_

**Figure 7:**
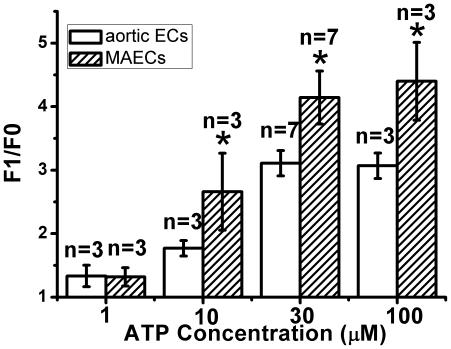
ATP-induced store Ca^2+^ release in endothelial cells in the absence of H_2_O_2_ pretreatment. Shown were the maximal [Ca^2+^]_i_ changes to different concentration of ATP (1 µM, 10 µM, 30 µM, 100 µM) in aortic ECs and MAECs as expressed in F1/F0. Cells were bathed in 0Ca^2+^-PSS in the absence of H_2_O_2_ pretreatment. Mean ± SEM of 3 to 7 independent experiments (10 to 15 cells per experiment). *, *P*<0.05 as compared to aortic ECs.

We also compared ATP-induced Ca^2+^ store release in aortic ECs and MAECs in the absence of H_2_O_2_ pretreatment. Cells bathed in a nominal Ca^2+^-free solution were challenged with different concentrations of ATP. In both cell types, ATP evoked [Ca^2+^]_i_ rises in a concentration dependent manner (Figure 7). Furthermore, the [Ca^2+^]_i_ response in MAECs was more sensitive to ATP than that in aortic ECs (Figure 7).

### Non-involvement of hydroxyl radical

**Figure 8:**
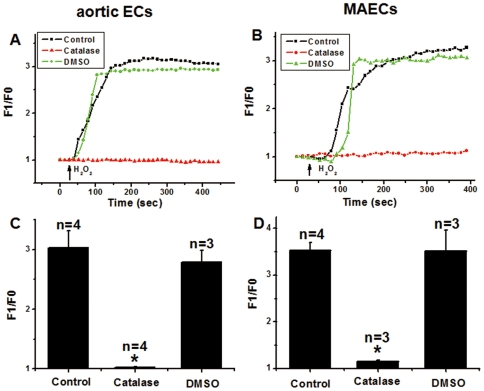
Effect of catalase and DMSO on H_2_O_2_-induced [Ca^2+^]_i_ rises in aortic ECs and MAECs. **A and B**. Representative traces of H_2_O_2_-induced [Ca^2+^]_i_ rises in the presence or absence of catalase or DMSO in N-PSS. 2000 U/ml catalase or 2% DMSO was added 30 min prior to the addition of H_2_O_2_ (5 mM). Fluorescence intensity before application of H_2_O_2_ was normalized to 1 as F0. **C and D**. Summary of data showing the effect of 2000 U/ml catalase and 2% DMSO treatment on H_2_O_2_-induced maximal [Ca^2+^]_i_ rises in aortic ECs (C) and MAECs (D) as expressed in F1/F0. Mean±SEM of 3 to 4 independent experiments (10 to 15 cells per experiment). *, *P*<0.05 as compared to control.

The effect of H_2_O_2_ on [Ca^2+^]_i_ could result from the action of H_2_O_2_ itself or from its metabolic product hydroxyl radical. Catalase was used to remove H_2_O_2_ and DMSO was used to scavenge hydroxyl radical. Pretreatment of cells with 2000 U/ml catalase for 30 min abolished the H_2_O_2_-induced [Ca^2+^]_i_ rises in both types of endothelial cells, whereas 2% DMSO had no effect (Figure 8A–8D). Our data suggest hydroxyl radical was not involved in the H_2_O_2_-induced [Ca^2+^]_i_ rises in both types of endothelial cells.

### HX-XO-induced [Ca^2+^]_i_ rises were caused by superoxide anion and hydrogen peroxide

**Figure 9:**
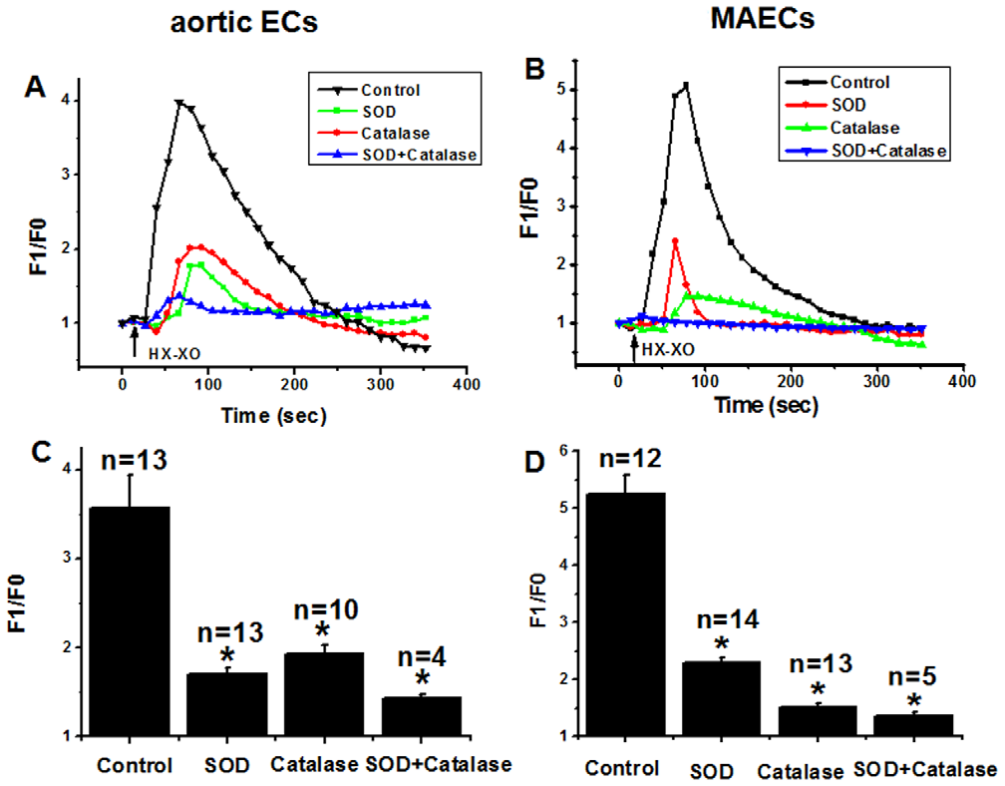
Effect of SOD and catalase on HX-XO-induced [Ca^2+^]_i_ rises in aortic ECs and MAECs. **A and B**. Representative traces of the [Ca^2+^]_i_ in response to HX-XO (200 µM HX; 20 mU/ml XO). The cells were pre-treated with or without 250 U/ml SOD for 20 min or 2000 U/ml catalase for 30 min prior to the addition of HX-XO in N-PSS. Fluorescence intensity before HX-XO application was normalized to 1 as F0. **C and D**. Summary of data showing the effect of SOD (250 U/ml, 20 min pre-treatment) or catalase (2000 U/ml, 30 min pretreatment) or both agents on HX-XO-induced maximal [Ca^2+^]_i_ rises in aortic ECs (C) and MAECs (D) as expressed in F1/F0. Mean±SEM of 4–13 independent experiments (10 to 15 cells per experiment). *, *P*<0.05 as compared to control.

Effect of HX-XO on [Ca^2+^]_i_ was also studied. HX-XO reacts to yield superoxide anions, which may spontaneously or enzymatically dismutate into H_2_O_2_ [4]. Application of HX-XO (200 µM and 20 mU/ml, respectively) evoked rapid [Ca^2+^]_i_ rises in both types of endothelial cells. Pre-incubation of the cells for 20 min with 250 U/ml superoxide dismutase (SOD), an enzyme that causes superoxide dismutation, reduced the [Ca^2+^]_i_ rises (Figure 9A–9D). Pretreatment with catalase (2000 U/ml, 30 min) also reduced the HX-XO-induced [Ca^2+^]_i_ rises (Figure 9A–9D). Catalase had a larger effect on the HX-XO-induced [Ca^2+^]_i_ responses in MAECs (reduction by 71±0%, n = 13) than in aortic ECs (reduction by 47±0%, n = 10) (Figure 9C and 9D). Combined treatment of SOD and catalase almost completely abolished the HX-XO-induced [Ca^2+^]_i_ rises in both types of endothelial cells (Figure 9A–9D).

## Discussion

[Ca^2+^]_i_ change is an important early signal for ROS-induced endothelial function and dysfunction. However, only a few studies have investigated ROS-induced Ca^2+^ signaling in the endothelial cells derived from small-sized arteries [8], [19] and it is unclear whether there is any difference in ROS-induced [Ca^2+^]_i_ responses in endothelial cells from different-sized arteries. In the present study, we compared the effect of H_2_O_2_ on [Ca^2+^]_i_ in endothelial cells from large-sized arteries and small-sized arteries. The results show that H_2_O_2_ stimulated [Ca^2+^]_i_ rises in both cell types. The H_2_O_2_-induced [Ca^2+^]_i_ rises could be blocked by U73122 and XeC, suggesting that the signaling cascade involves phospholiase C activity, IP_3_ production, and Ca^2+^ release through IP_3_ receptors. The increased IP_3_ production following H_2_O_2_ treatment was confirmed by IP_3_ measurement. It is well documented that Ca^2+^ release from IP_3_-sensitive Ca^2+^ stores would stimulate Ca^2+^ influx through store-operated Ca^2+^ entry mechanism [20]. Indeed, we found that H_2_O_2_ treatment could enhance Ca^2+^ entry when the bath solution contained Ca^2+^.

There are conflicts in reports as to how ROS treatment would affect the [Ca^2+^]_i_ responses to subsequent agonist challenge in endothelial cells [7], [9], [12], [15]. In some studies, H_2_O_2_ and superoxide anions were found to reduce the agonist-induced [Ca^2+^]_i_ rises [9], [15]. In other studies, ROS treatment was found to enhance [9], [12] or have no effect on the agonist-induced [Ca^2+^]_i_ responses [7]. In the present study, we found that H_2_O_2_ treatment reduced the [Ca^2+^]_i_ responses to ATP in H_2_O_2_ concentration-dependent and H_2_O_2_ incubation time-dependent manners in mouse aortic ECs and MAECs. The reduced [Ca^2+^]_i_ responses to ATP were due to a pre-depletion of intracellular Ca^2+^ stores during H_2_O_2_ treatment. Two lines of evidence support this: 1) After H_2_O_2_ treatment, the store Ca^2+^ release in response to ATP became much smaller. 2) Direct measurement of store Ca^2+^ content by Mag-fluo4 demonstrated a reduction in store Ca^2+^ content after H_2_O_2_ treatment. Interestingly, our data clearly indicate that endothelial cells from small-sized arteries (MAECs) were more sensitive to H_2_O_2_ treatment than those of large-sized arteries (aortic ECs) with regard to their store Ca^2+^ release and subsequent [Ca^2+^]_i_ responses to ATP. This type of differential sensitivity/response of store Ca^2+^ release to ROS treatment could explain some data conflicts in the literature. For example, Volk et al., reported that, in rat liver artery endothelial cells, ROS treatment had no effect on the [Ca^2+^]_i_ responses to subsequent ATP or histamine challenge [7]. But they used a relatively low concentration of ROS [7]. It is possible that such a low concentration of ROS might not be sufficient to cause marked store Ca^2+^ depletion. As a result, no change in [Ca^2+^]_i_ responses to agonists would be expected.

What could be the underlying cellular mechanism for the higher sensitivity of [Ca^2+^]_i_ responses to H_2_O_2_ in MAECs than in aortic ECs? H_2_O_2_-induced IP_3_ production was similar in MAECs and aortic ECs, therefore IP_3_ production was not the reason. Alternatively, this could be due to more abundant IP_3_ receptor expression and/or a higher IP_3_ receptor sensitivity to IP_3_ in MAECs than in aortic ECs. If this is true, [Ca^2+^]_i_ responses to other agonists is also expected to be higher in MAECs than in aortic ECs. Indeed, we found that similar high sensitivity of intracellular store Ca^2+^ release to ATP in MAECs than in aortic ECs (Figure 7). Therefore, we speculate that MAECs may express more IP_3_ receptors and/or the sensitivity of IP_3_ receptors to IP_3_ may be higher in MAECs than in aortic ECs.

The higher sensitivity of [Ca^2+^]_i_ responses to H_2_O_2_ in the endothelial cell of small-sized arteries could have physiological and/or pathological implication. At physiological concentration, H_2_O_2_ is a vasodilator and it causes endothelium-dependent and endothelium-independent vascular dilation [3], [23], [24]. The effect of H_2_O_2_ as a vascular dilator is often found in small-sized arteries and arterioles [3], [25]. In contrast, in large-sized arteries nitric acid is a more important vascular dilator [26]. Because [Ca^2+^]_i_ rises endothelial cells often trigger vascular dilation, a more sensitive [Ca^2+^]_i_ response to H_2_O_2_ in endothelial cells would allow H_2_O_2_ to serve as a more effective vascular dilator in small-sized arteries and arterioles. On the other hand, a high [Ca^2+^]_i_ sensitivity to H_2_O_2_ could also have pathological consequence. Excessive Ca^2+^ accumulation may lead to endothelial cell apoptosis and cell death [4]. Therefore, it is possible that endothelial cells in small-sized arteries or arterioles might be more vulnerable to ROS-induced cell damage.

H_2_O_2_ can be converted to hydroxyl radical in the presence of Fe^2+^ [4]. However, in the present study the effect of H_2_O_2_ on [Ca^2+^]_i_ rises in endothelial cells could not be attributed to hydroxyl radical, because the H_2_O_2_ effect was not affected by DMSO, which is an efficient hydroxyl radical scavenger [21]. In contrast, H_2_O_2_ effect was abolished by catalase, which converts H_2_O_2_ to O_2_ and H_2_O, suggesting a direct action of H_2_O_2_. We also investigated the effect of HX-XO on [Ca^2+^]_i_ in mouse aortic ECs and MAECs. HX-XO is one of most widely used methods to generate superoxide anions, which may in turn dismutate into H_2_O_2_ spontaneously or enzymatically [4]. We found that the HX-XO-induced [Ca^2+^]_i_ rises could be attributed to involvement of superoxide anions and H_2_O_2_ but not hydroxyl radicals in both types of endothelial cells, because the response was reduced by SOD and catalase but not by DMSO. There were relatively more H_2_O_2_ contribution in HX-XO-induced [Ca^2+^]_i_ rises in endothelial cells of small-sized arteries (MAECs) than in those of large-sized arteries (aortic ECs). Previously, different reports have claimed different ROS, including H_2_O_2_ [5], [7], [10], hydroxyl radical [10], and/or superoxide anions [5], [10], to be the contributing factors that were involved in HX-XO provoked-[Ca^2+^]_i_ rises in endothelial cells. The discrepancy in results could be due to a variety of factors including endothelial cell sources and/or culture conditions.

In conclusion, we found both Ca^2+^ entry and store Ca^2+^ release contributed to the H_2_O_2_-induced [Ca^2+^]_i_ rises in endothelial cells. H_2_O_2_ treatment depleted the intracellular Ca^2+^ stores, resulting in reduced [Ca^2+^]_i_ responses to subsequent agonist challenge. The store Ca^2+^ release and subsequent [Ca^2+^]_i_ responses to ATP were more sensitive to H_2_O_2_ treatment in endothelial cells of small-sized arteries than those of large-sized arteries. This study highlights the similarity and difference of ROS-induced [Ca^2+^]_i_ responses in endothelial cells from large-sized arteries and small-sized arteries.

## Methods

### Ethics statement

We followed Guide for Animal Care and Use of Laboratory Animals published by the US National Institute of Health. The protocols for animal experiments were approved by Animal Experimentation Ethics Committee, The Chinese University of Hong Kong (approval number# 09/060/MIS).

### Primary Cell Culture

Animals were supplied by the Laboratory Animal Service Center of the Chinese University of Hong Kong (Hong Kong, China). We followed Guide for Animal Care and Use of Laboratory Animals published by the US National Institute of Health. The protocols for animal experiments were approved by Animal Experimentation Ethics Committee, The Chinese University of Hong Kong (approval number# 09/060/MIS). Male C57 mice (8–12 weeks) were sacrificed by inhalation of CO_2_. Primary cultured aortic endothelial cells (aortic ECs) and mesenteric artery endothelial cells (MAECs) were dissociated from mouse aorta and mesenteric arteries of the first to tertiary branches (internal diameter = 60–200 µm), respectively, using the methods described elsewhere [27]. Aortic ECs and MAECs were cultured in endothelial cell growth medium supplemented with 1% bovine brain extract.

### [Ca^2+^]_i_ Measurement

Cells were prepared and loaded with a membrane permeant fluorescence dye Fluo4/AM (Molecular Probes, Inc., NJ) for observing their [Ca^2+^]_i_ responses to H_2_O_2_ or HX-XO or ATP. Briefly, the cells were seeded on circular glass discs at 37°C overnight supplemented with the culture medium. For the fluorescence dye loading, cells were incubated for 1 hr in dark at room temperature with 10 µM Fluo4/AM and 0.02% Pluronic acid F-127 in normal physiological saline solution (N-PSS), which contained in mM: 1 CaCl_2_, 140 NaCl, 1 KCl, 1 MgCl_2_, 10 glucose, and 5 Hepes at pH 7.4. The circular discs containing the endothelial cells were then pinned in a specially designed chamber. The chamber was placed on the stage of an inverted microscope (Nikon Diaphot 200). During experiments, cells were bathed in N-PSS or 0.5Ca^2+^-PSS or 0Ca^2+^-PSS. The composition of 0.5Ca^2+^-PSS and 0Ca^2+^-PSS was similar to N-PSS except for Ca^2+^ concentration (0.5 mM CaCl_2_ for 0.5Ca^2+^-PSS, and nominal Ca^2+^-free for 0Ca^2+^-PSS). All agents were applied directly to the bath along the side of the chamber. Solutions were then mixed by pipetting gently up and down for a few times. Experiments were performed at room temperature. Fluorescence signals were recorded by MRC-1000 Laser Scanning Confocal Imaging System with MRC-1000 software (Bio-Rad) with the excitation wavelength of 488 nm and a 515 nm-long pass emission filter. The Ca^2+^ responses were displayed as the ratio of fluorescence relative to the intensity before H_2_O_2_ or ATP or HX-XO (F1/F0). Due to variation in [Ca^2+^]_i_ responses between different batches of cells, each series of experiments had its own control.

### Measuring Ca^2+^ Content in Intracellular Ca^2+^ Stores

Cells were loaded with fluorescence dye Mag-fluo4/AM (Molecular Probes, Inc., NJ) for observing the Ca^2+^ level in intracellular Ca^2+^ stores. Briefly, cells were seeded on circular glass plates at 37°C overnight supplemented with the culture medium. As for the fluorescence dye loading, cells were incubated with 5 µM Mag-fluo4/AM in dark at 37°C for 45 min, and 0.02% Pluronic acid F-127 in N-PSS. Cells were then washed with the indicator-free N-PSS and incubated at 37°C for 45 min to unload the Mag-fluo4 from cytoplasm. The circular discs containing the endothelial cells were then pinned down in a specially designed chamber. The chamber was placed on the stage of an inverted microscope (Nikon Diaphot 200). Mag-fluo4 fluorescence was recorded by MRC-1000 Laser Scanning Confocal Imaging System with MRC-1000 software (Bio-Rad) with the excitation wavelength of 488 nm and a 515 nm-long pass emission filter. The cells were then treated with or without H_2_O_2_ for 30 minutes. Because Mag-fluo4 fluorescence was reported to be light-sensitive and could be quenched by light exposure, laser emission to samples was cut off during the period of H_2_O_2_ treatment. Fluorescence signals were then collected before and after 30-minute H_2_O_2_ treatment. The change in store Ca^2+^ content is displayed as Mag-fluo4 intensity change in percentage.

### IP_3_ measurement

The amount of IP_3_ was measured using HitHunter™ IP_3_ Assay Fluorescence Polarization Detection-Green Kits (DiscoveRx Tech, Fremont, CA, USA), a reliable and convenient methodology based on competitive binding between an IP_3_ fluorescence tracer and unlabeled IP_3_ from the cell lysates or standards. Free IP_3_ competes at the IP_3_ binding protein and allows the IP_3_ tracer to rotate freely upon excitation with plane polarized light. The polarized signal is inversely proportional to the amount of the free unlabelled IP_3_. Thus, polarization signal is decreased when the concentration of free IP_3_ is increased [22]. Briefly, aortic ECs and MAECs were treated with different concentrations of H_2_O_2_ (500 µM, 2 mM, 5 mM) for 5 min in black 96-well plates. The cellular reactions were terminated by placing cells on ice followed by addition of 0.2 N perchloric acid to lyse the cells. The plate was then shaken at 650 rpm for 5 min. The IP_3_ tracer was subsequently added to each well, followed by IP_3_ binding protein. The polarized fluorescence from the IP_3_ tracer (fluorescein) was read using a Wallac EnVision™ Microplate Reader (Perkin Elmer, Wallac, EnVision, Finland) with a polarization mirror, a 485 nm excitation filter and a 530 nm emission filter. IP_3_ concentration was calculated from the IP_3_ standard curve and expressed as pmole/1×10^6^ cells.

### Data Analysis

Data Analysis was performed with Software Confocal Assistant and Metafluor. All representative traces were plotted by using Prism 3.0 (GraphPad, San Diego, CA, USA). Summarized data were expressed as the mean±SEM and analyzed with two-tailed Student's t test at a p<0.05 level of significance.
